# “Though helpful, still hesitant”: a TAM-based qualitative study on older adults’ ambivalent acceptance and model extensions in AI fitness coaches

**DOI:** 10.3389/fpsyg.2025.1666755

**Published:** 2025-12-17

**Authors:** Yi Yau, Ya Chun Shen

**Affiliations:** Department of Leisure and Recreation Management, Taipei City University of Science and Technology, Taipei, Taiwan

**Keywords:** ambivalent acceptance, digital health, emotional, human pose estimation, human–AI interactions

## Abstract

**Background:**

With the deepening integration of artificial intelligence (AI) and health promotion strategies, digital health applications have continued to expand. AI fitness coaches have emerged as promising tools to support physical activity among older adults. Although models such as the Unified Theory of Acceptance and Use of Technology and the Senior Technology Acceptance Model have extended the Technology Acceptance Model (TAM) by introducing external factors across technological, psychological, and socio-emotional domains, further research is needed to contextualize these variables within AI-based environments. Prior studies have focused mainly on general digital or assistive technologies, with limited attention to emotionally and cognitively complex human–AI interactions such as AI fitness coaching. Therefore, this study adopts TAM as the core theoretical framework, as its concise structure suits exploratory interviews while preserving participants’ lived experience richness.

**Methods:**

Participants were recruited through public announcements at sports and neighborhood activity centers in Northern Taiwan, with support from community leaders. This qualitative study used TAM as a foundational framework and conducted semi-structured interviews with 12 older adult participants. The interview protocol explored their interactions with AI fitness coaches, emphasizing perceived usefulness, ease of use, emotional responses, and contextual influences. Verbatim transcripts were thematically analyzed using both deductive codes from TAM and inductive codes to capture emerging themes. Two researchers independently coded the data and reached consensus to ensure analytic rigor.

**Results:**

Perceived usefulness and ease of use were shaped not only by system design but also by aging-related factors such as physical limitations, life history, and autonomy. While participants expressed cognitive approval of AI fitness coaches, emotional resistance revealed ambivalent acceptance. Findings highlight that emotional, social, and cultural factors–often overlooked in TAM–play a significant role in shaping older adults’ technology engagement.

**Conclusion:**

This study expands TAM by emphasizing that technology acceptance is a dynamic, context-sensitive process shaped by emotional and cultural influences. It underscores the need to adapt external variables to specific settings and encourages designing AI fitness technologies that foster emotional resonance and cultural relevance. The concept of “ambivalent acceptance” offers new insight into how aging individuals engage with technology, contributing to both theoretical development and inclusive design.

## 1 Introduction

With the rapid advancement of artificial intelligence (AI) technologies, AI-based virtual fitness coaching systems have increasingly been applied in the domains of health promotion and home-based exercise (Culos-Reed et al., 2021). In aging societies, these systems are regarded as vital tools to support older adults in managing their health autonomously (El Kamali et al., 2020). According to the World Health Organization (2024), the global population aged 60 and above is projected to reach 2.1 billion by 2050. Concurrently, health expenditures related to aging are rising sharply, underscoring the urgent need for AI-enabled tools to facilitate self-health management among older adults.

Commonly referred to as “AI fitness coaches,” these systems utilize real-time feedback, posture recognition, and personalized recommendations to enhance users’ awareness of physical activity, boost motivation, and promote sustained engagement in health behaviors (Kettunen et al., 2024). For older adults experiencing functional decline, AI fitness coaches offer a highly accessible (Shen et al., 2025; Tsai et al., 2025), personalized (Shen et al., 2025; Tsai et al., 2025), and sustainable means of maintaining physical capability (Shen et al., 2025; Meng et al., 2022; Tsai et al., 2025), preventing falls (Tsai et al., 2025; Wong et al., 2023), and delaying physiological deterioration (Shen et al., 2025; Tsai et al., 2025; Wong et al., 2023). Ultimately, such technologies contribute to improved independence and quality of life (El Kamali et al., 2020). Furthermore, the use of digital technologies in promoting social well-being has been shown to reduce social isolation among the older adults (Sen et al., 2022). As demonstrated by Fang et al. (2025), AI-enhanced physical activity interventions can significantly improve mental health in older adults by promoting emotional regulation, cognitive recovery, and psychological resilience through personalized support and adaptive exercise monitoring. These findings highlight AI’s potential to strengthen intrinsic motivation and self-efficacy–key psychological mechanisms for sustaining well-being in aging populations.

Contemporary AI fitness coaches are primarily developed based on human pose estimation frameworks such as MediaPipe, OpenPose, AlphaPose and DensePose (Edriss et al., 2025). OpenPose is an open-source framework developed by Carnegie Mellon University that detects 25 body keypoints in real time; it supports multi-person tracking and biomechanical analysis, making it widely used in sports performance and posture assessment, though it is sensitive to lighting conditions and occlusion; MediaPipe, developed by Google, incorporates the BlazePose module to detect 33 keypoints and is optimized for lightweight, real-time applications such as mobile computing; it offers high efficiency and accuracy but does not support multi-person detection; AlphaPose, created by the Chinese University of Hong Kong, also detects 25 keypoints and excels in action classification and gait analysis, especially in sequence-based motion recognition, though it tends to exhibit higher variability in joint range of motion and measurement error; DensePose, developed by Facebook AI, maps every pixel of the human body onto a 3D surface mesh, enabling high-resolution full-body reconstruction for virtual and augmented reality; it supports multi-person tracking but requires substantial computational resources and technical expertise (Edriss et al., 2025). Each of the four frameworks has its own advantages and limitations; therefore, selecting the appropriate one should depend on the specific task requirements, computational resources, and application context. These systems integrate voice interfaces and real-time visual feedback mechanisms to provide exercise guidance, posture correction, and risk warnings. They are characterized by high accessibility and low usability barriers (Huang Z. et al., 2024; Yu et al., 2021), and have been widely adopted in fitness centers, home environments, community programs, and long-term care facilities (Gowrish et al., 2024).

However, older adults often exhibit ambivalent attitudes when engaging with AI fitness coaches, simultaneously harboring both positive expectations and negative concerns. While some users appreciate the systems for their personalized feedback and real-time responsiveness, others report feelings of emotional detachment or a sense of interactional coldness, which contributes to a lack of trust (Wong et al., 2025). Some older adults express favorable views toward monitoring technologies, such as perceiving them as tools that enhance safety, yet at the same time they experience concerns regarding privacy invasion, reduced autonomy, and a perceived loss of control. This combination clearly reveals a sense of ambivalence (Boström et al., 2013). These psychological conflicts and contextual complexities present challenges to existing Technology Acceptance Models. The Technology Acceptance Model (TAM), introduced by Davis (1989), focuses on two primary constructs: Perceived Usefulness (PU) and Perceived Ease of Use (PEOU), which are used to explain users’ attitudes and behavioral intentions. In an effort to unify multiple fragmented theories of technology acceptance, Venkatesh et al. (2003) proposed the Unified Theory of Acceptance and Use of Technology (UTAUT), which emphasizes rational and structural determinants such as performance expectancy, effort expectancy, social influence, and facilitating conditions. Nevertheless, as Kenesei et al. (2025) have pointed out, despite its strong predictive capabilities, UTAUT provides limited consideration of psychological factors such as emotion, thereby reducing its ability to fully account for the emotional dimensions of human–technology interaction. To address the limitations of TAM and UTAUT in the context of aging populations, Chen and Chan (2014) proposed the Senior Technology Acceptance Model (STAM), which incorporates external variables such as gerontechnology self-efficacy, technology anxiety, perceived health status, and social relationships to provide a more comprehensive explanatory framework. While STAM offers strong theoretical alignment with aging-related research, its complex structure and predominant use in large-scale quantitative studies make it less suitable for capturing nuanced subjective experiences and contextual interactions. This study adopts a semi-structured interview design aimed at deeply exploring older adults’ subjective experiences and emotional responses during interactions with AI fitness coaches. Recent critiques of gerontechnology research highlight an overemphasis on “technological intervention,” advocating instead for a more relational approach that examines how aging and technology co-construct one another. Scholars call for greater attention to contextual, relational, and interactive dimensions rather than viewing technology as a one-way solution for aging-related issues (Peine and Neven, 2019).

Given TAM’s relatively concise structure and its widespread use in exploratory research, it was selected as the foundational theoretical framework. TAM allows for a focused examination of PU and PEOU and facilitates meaningful linkage to participants’ narrative accounts. Furthermore, a systematic review by Yau and Hsiao (2022) on TAM and older adults’ use of exercise technologies identified trust, emotional interaction, and social support as critical external variables influencing technology adoption among older users. This finding reinforces TAM’s potential for theoretical extension while highlighting the limitations of traditional models in explaining emotionally and socially rich contexts like AI-based fitness applications. As emphasized by Wong et al. (2025) and Boström et al. (2013), older adults do not simply “accept” or “reject” technologies; rather, they remain suspended between expectation and doubt. As suggested by Ambivalence Theory, individuals may experience both attraction and hesitation toward new technologies, reflecting an inner tension between perceived benefits and emotional resistance (Kaplan, 1972; Thompson et al., 1995). This ambivalent stance illustrates why traditional adoption models such as the Technology Acceptance Model may be insufficient when they focus solely on perceived usefulness and perceived ease of use. A purely binary interpretation of “use or non-use” overlooks the affective complexity and relational nature of older adults’ interactions with technology. Consequently, qualitative inquiry is required to uncover the emotional undercurrents and psychological tensions that shape these acceptance experiences. In sum, while TAM provides a foundational lens for understanding older adults’ technology adoption processes, its original structure falls short of capturing the dynamic psychological and emotional processes involved in using AI fitness coaches. Against this backdrop, the present study adopts TAM as the theoretical framework and employs qualitative interviews to explore the lived experiences of older adults using AI fitness coach systems. Particular emphasis is placed on understanding the coexistence of positive motivations and negative emotional responses, and identifying extended constructs that may influence technology adoption.

Against this backdrop, the present study adopts TAM as the theoretical framework and employs qualitative interviews to explore the lived experiences of older adults using AI fitness coaches systems. Particular emphasis is placed on understanding the coexistence of positive motivations and negative emotional responses, and identifying extended constructs that may influence technology adoption. The objectives of this study are threefold: (1) to examine the applicability and limitations of the TAM framework in the context of older adult users; (2) to analyze the psychological mechanisms underlying technology acceptance in the presence of emotional ambivalence; and (3) to identify emergent external variables that may inform potential extensions of the TAM model. This study not only contributes to the theoretical expansion of TAM but also offers empirical insights for the design of emotionally responsive and user-centered AI fitness coaches systems, thereby informing the development of age-friendly technologies and related policy initiatives.

## 2 Literature review

### 2.1 Applicability and extensions of the Technology Acceptance Model (TAM) in the context of older adults

Technology Acceptance Model proposed by Davis et al. (1989), posits that perceived usefulness (PU) and perceived ease of use (PEOU) jointly influence users’ attitudes toward use (ATT) a technology, which subsequently affects their behavioral intention to use (BI) and actual usage behavior. Due to its theoretical clarity and strong explanatory power, TAM has been widely adopted in fields such as education (Granić and Marangunić, 2019), healthcare, and mobile technology adoption (Rahimi et al., 2018).

However, as the application contexts of technology grow increasingly diverse, the explanatory power of TAM has been called into question, particularly in relation to older adults’ interaction with emerging technologies such as AI-based fitness coaches (Yang et al., 2025). Studies have pointed out that TAM’s original constructs fail to account for critical age-related factors such as emotional needs, trust formation, sense of control, and challenges in social interaction. Moreover, the model lacks mechanisms for addressing negative psychological responses common among older adults, including frustration and feelings of alienation (Wong et al., 2025). These limitations suggest that older adults’ technology acceptance processes are more complex and multidimensional than those of the general population, rendering PU and PEOU insufficient for comprehensively predicting behavioral outcomes.

A systematic review by Yau and Hsiao (2022) on older adults’ use of sports technology revealed that, among 18 identified influencing factors, only three corresponded to TAM’s original constructs. The remaining 15 were classified as external variables, encompassing psychosocial dimensions such as trust, emotional connectedness, self-efficacy, digital literacy, and social support.

To address these limitations and enhance the applicability of TAM in aging contexts, the present study adopts TAM as a foundational framework for interview design, while incorporating three representative theoretical sources to guide the classification and integration of external variables: (1) the extended variables identified by Yau and Hsiao (2022); (2) the UTAUT proposed by Venkatesh et al. (2003); and (3) the gerontechnology framework developed by Czaja et al. (2006). Based on functional attributes and underlying psychological mechanisms, these external variables are categorized into three key domains: (1) device and technological factors, (2) psychological and cognitive factors, and (3) social and emotional factors.

This classification not only complements the original TAM framework but also provides a more nuanced understanding of the acceptance process and potential barriers faced by older adults when using AI-based fitness coaches systems. In doing so, it offers practical and human-centered recommendations for system design and implementation strategies aimed at enhancing age-friendly technology adoption.

### 2.2 Impacts of AI-driven fitness coaching systems on the health and care of older adults

In recent years, AI-driven fitness coaching systems have demonstrated increasing transformative potential in the field of older adults’ health promotion, evolving from simple exercise support tools into integrated smart care solutions that encompass exercise prescription, emotional support, social interaction, and health monitoring. As noted in a systematic review by Shen et al. (2025), socially assistive AI fitness robots such as Pepper and NAO not only adjust exercise intensity and pacing according to individual needs but also provide real-time verbal feedback and behavioral prompts. These systems incorporate affective simulation and companionship features, which significantly enhance exercise participation, sleep quality, and subjective well-being among older adults, particularly those experiencing social isolation, cognitive decline, or low motivation. These findings highlight the critical role of anthropomorphic interaction mechanisms in facilitating behavioral change.

Further evidence from a randomized controlled trial by Meng et al. (2022) showed that a hybrid training program combining eight-form Tai Chi with strength and endurance training (TCSE) was the most effective in improving gait speed and cardiorespiratory endurance in frail older adults. The study also introduced an explainable AI (XAI) model to accurately predict post-intervention outcomes based on baseline physical fitness and intervention type, underscoring the dual value of AI coaches as both training facilitators and clinical decision-support tools. This approach advances the development of personalized, dynamically adaptive exercise prescriptions for aging populations.

In community settings, Tsai et al. (2025), using a mixed-methods design, found that intelligent exercise systems significantly improved older adults’ digital resilience and self-efficacy in health management. Through user-friendly interfaces and real-time feedback, these systems fostered continuous engagement and learning. Technological internalization, personalized exercise experience, and peer support were identified as three key themes essential to emotional regulation and social engagement. These findings align with the TAM, emphasizing perceived usefulness and ease of use as mediating factors in sustained technology adoption among older adults.

From a macro perspective, Fang et al. (2025) conducted a bibliometric analysis of 1,831 studies and synthesized the mechanisms through which AI-enhanced fitness coaching contributes to mental health promotion. Identified pathways include: (1) upregulation of neurotrophic factors such as BDNF and GDNF, (2) emotional and physiological monitoring, (3) personalized goal setting with real-time behavioral feedback, and (4) digital social interaction and companionship. These systemic interventions not only mitigate depression and anxiety and enhance cognitive and behavioral functioning, but also provide deeper support for autonomy, purpose, and social belonging.

Overall, AI fitness coaching systems are shaping a multidimensional smart health paradigm that integrates motion monitoring, emotional support, cognitive reinforcement, and social empowerment. They play an increasingly vital role not only in maintaining and restoring physical function in older adults, but also in enhancing mental well-being, autonomous decision-making, digital literacy, and active engagement with technology.

### 2.3 Emotional ambivalence and acceptance challenges of AI fitness coaches among older adults

Previous studies have shown that older adults often exhibit ambivalent psychological reactions when encountering new technologies. On the one hand, they recognize the potential benefits of technological innovations, such as convenience, monitoring capabilities, and health promotion (Kettunen et al., 2024). On the other hand, empirical research indicates that older adults frequently report anxiety and resistance related to technology use, often attributed to operational difficulties, privacy concerns, cognitive load in learning new systems, and a lack of emotional interaction (Peek et al., 2014). These barriers shape the trajectory of technology acceptance among older adults, whose engagement often oscillates between enthusiasm and hesitation. The complex interplay between acceptance and resistance suggests that technology adoption among older adults is a dynamic, iterative, and non-linear decision-making process.

An increasing body of research has pointed out that older adults’ engagement with AI-driven health technologies, including AI-based fitness coaching systems, is not determined by a binary view of “acceptance or rejection” but is instead governed by emotional ambivalence that is deeply rooted in competing socio-psychological values. This ambivalence stems from the dual evaluation of technology: while older adults acknowledge the functional benefits of AI, including self-monitoring, chronic disease management, and personalized care, they also experience anxiety, distrust, and emotional discomfort (Wong et al., 2025). When AI systems are perceived as cold, dehumanized, or replacing human care rather than supporting it, such emotional contradictions are further amplified. Correspondingly, Boström et al. (2013) identified three interrelated axes of tension: autonomy versus safety, privacy versus surveillance, and self-interest versus social expectations. Each of these reflects the underlying desire among older adults to maintain control and preserve their identity in later life. These tensions highlight the psychological strain that emerges when technology intervenes in the aging process. These tensions highlight the psychological strain that emerges when technology intervenes in the aging process.

Ho et al. (2023) found that older Japanese clinic visitors expressed not only appreciation for the efficiency of emotional AI in healthcare but also a deep sense of unease, especially regarding loss of control and a lack of affective connection with AI systems. This emotional tension led to indecision and hesitance toward full-scale adoption, revealing that technology acceptance is not merely a rational process, but one shaped by emotional needs and psychological discomforts.

Similarly, Rony et al. (2025), in a qualitative study conducted in Bangladesh, reported strong emotional resistance toward AI-assisted medical decision-making. Despite recognizing the potential benefits of AI in improving decision accuracy and care efficiency, many older patients expressed concerns over dehumanization, lack of empathy, threats to data privacy, and insufficient informed consent. These anxieties mirror the ambivalent pattern observed in the present study, where older adults perceived AI fitness coaches as helpful yet insufficiently trustworthy, often failing to provide the human warmth, communication clarity, and autonomy that they valued.

Wong et al. (2025) substantiated these findings by employing the Theoretical Domains Framework (TDF) and the COM-B model to examine the perspectives of older adults in Hong Kong regarding AI-driven health technologies. Their study revealed that emotional uncertainty, low perceived competence, and fears of data misuse or scams strongly influenced acceptance patterns. Importantly, older adults preferred positioning AI as a supportive partner rather than a replacement for human interaction, emphasizing the irreplaceable value of relational warmth and collaborative agency in health contexts.

These tensions highlight the psychological strain that emerges when technology intervenes in the aging process. This phenomenon echoes Kaplan’s (1972) theoretical proposition that ambivalence is not a sign of indecision but rather a manifestation of simultaneous positive and negative evaluations toward the same object–resulting in conditional or fluctuating engagement. Similarly, Thompson et al. (1995) argued that ambivalence is not noise in attitude measurement but a meaningful construct that deserves recognition as a central component of human behavior. In the context of technology adoption among older adults, Chen and Chan (2011) observed a similar pattern: although seniors acknowledge the utility and convenience of technology, they simultaneously doubt their own competence, worry about operational complexity, and fear privacy risks. Consequently, their attitudes tend to be both accepting and detached, reflecting a mixed emotional stance. This aligns with Inder et al.’s (2010) notion of ambivalent acceptance, emphasizing that older adults’ responses to new technologies are not simply characterized by rejection or denial but represent a dynamic psychological process situated between trust and skepticism.

In the case of AI-based fitness coaching systems, even when equipped with features such as real-time posture detection, voice guidance, and personalized adjustments, users may still experience negative emotional reactions if the interaction feels mechanical, emotionally detached, or fails to establish a sense of trust (Wong et al., 2025). Such perceptions often lead to user resistance and eventual discontinuation. Therefore, focusing solely on PU and PEOU is insufficient to fully explain older adults’ intentions to adopt or continue using such systems. Overall, older adults’ attitudes toward AI health technologies exhibit a dynamic pattern of partial use, intermittent use, or hesitant use.

Taken together, these findings call for a rethinking of the Technology Acceptance Model (TAM) within aging contexts. Beyond PU and PEOU, essential constructs such as affective trust, perceived behavioral control, social validation, and ethical transparency must be incorporated to address the emotional complexity, cultural expectations, and human-AI relational dynamics experienced by older users.

This study argues that, beyond exploring the positive motivations and benefits associated with AI technologies, it is equally important to examine the emergence of negative emotions and resistance experiences. A comprehensive understanding of these dual emotional dynamics is crucial for clarifying the psychological contradictions and acceptance pressures older adults face when engaging with technology. These insights not only challenge the theoretical adequacy of the TAM in aging contexts but also lay a foundation for developing human-centered and emotionally attuned AI interaction designs, where systems achieve not only technical efficiency but also emotional resonance and psychological safety.

## 3 Materials and methods

### 3.1 Study design

In this qualitative study, we conducted semi-structured interviews with Taiwanese older adults in Mandarin to understand their perceptions of an AI fitness coach. During the formal interviews, participants first watched a 10-min video introducing the AI-based virtual fitness coach. They then engaged in a brief hands-on session, performing squat movements for 1 min guided by the system. This trial was identical in length and content for all participants to ensure consistency. Due to the COVID-19 pandemic at the time, prolonged physical contact was avoided, and the interactive component of this intervention was intentionally limited to reduce health risks. The entire process, which included explanation, video introduction, system trial, and interview, took approximately 1 h per participant. Prior to the start of this study, ethical approval was obtained from the Institutional Review Board, ensuring that all procedures complied with research ethics guidelines.

### 3.2 Recruitment

This study adopted a self-selection sampling method. Between August and November 2022, participants were recruited through public announcements at sports centers and neighborhood activity centers in Northern Taiwan, with the assistance of community leaders and local organizations. The research team also explained the study purpose and participation criteria to older adults on-site. The selection of Taipei City and New Taipei City as recruitment sites was based on both practical and research considerations. These highly urbanized areas have a dense population of older adults actively engaged in community health programs and offer well-developed digital infrastructure, public transportation, and access to health technology. Moreover, during the COVID-19 pandemic, their accessibility and healthcare readiness ensured a safe and efficient environment for conducting standardized interventions. As such, these locations provided both feasibility and relevance for studying urban older adults’ initial experiences with AI-driven fitness technologies.

### 3.3 Eligibility

The participants needed to meet the following criteria: (1) aged over 55 years, (2) in generally good physical health, and (3) had never used a virtual fitness coach.

### 3.4 Consent

This study was reviewed and approved by the Research Ethics Committee of National Taiwan Normal University, and was conducted in accordance with the ethical principles of the Declaration of Helsinki, ensuring informed consent, privacy protection, and research integrity for all participants. After determining eligibility, the overall study purpose, potential risks and benefits, and confidentiality measures were clearly explained to each participant. They were informed that participation was voluntary and that they could withdraw from the study at any time if they experienced discomfort or found any aspect unacceptable. The study did not begin until participants provided written informed consent by signing the consent form.

### 3.5 Data collection

Demographic data including age, gender, educational background, and experience using technology were collected, and semi-structured interviews were conducted with 12 participants. The Technology Acceptance Model was used to develop interview guidelines, including PEOU, PU, ATT and BI toward them. Table 1 shows the interview guidelines.

**TABLE 1:** Examples of interview questions using TAM.

No.	Interview questions	Dimension
1	How do you feel about using the AI fitness coaches platform? How about the buttons and video speed?	PEOU
2	Do you find the platform easy to use? Why?	PEOU
3	Is it easy to learn exercise routines on the platform? Why?	PEOU
4	Which is more convenient: using a virtual or a traditional coach? Why?	PEOU
5	Is the AI fitness coaches easy to operate for you? Why?	PEOU
6	Do you think the exercise education provided is important? Why?	PU
7	Do you find the AI fitness coaches important? Why?	PU
8	If the exercise education scores highest, what could be the reasons?	PU
9	Is the feedback from the monitoring system different from that of your friends? Why?	PU
10	Has the virtual coach been helpful to you? Why?	PU
11	Which is more effective: virtual or traditional exercise? Why?	PU
12	What are the differences between a traditional coach and a v AI fitness coaches? Which do you prefer, and why?	ATT
13	How does the encouragement from the virtual coach help you during exercise?	ATT
14	Does encouragement from the virtual coach make you want to exercise more? Why?	ATT
15	What’s the difference between regular use and frequent use for you? Why?	BI
16	Does having this AI fitness coaches make you want to exercise more? Why?	BI

Interviews were conducted by the first author, and all interviews were audio-recorded and subsequently transcribed verbatim by the first author to ensure consistency of transcription.

### 3.6 Data analysis

This study adopted a qualitative theoretical analysis approach to identify, analyze, and report themes emerging from the interview data. The first author, an assistant professor at a university, conducted the semi-structured interviews and had received formal training in research methodology, with expertise in health promotion among older adults and sports technology. To enhance the credibility of the data, all interviews were transcribed verbatim by the first author and subsequently member-checked by the participants to confirm the accuracy of meaning and content.

The second author, a graduate student in leisure and sport management, had completed coursework in qualitative research and possessed foundational skills in qualitative data analysis. After transcription, both the first and second authors independently conducted line-by-line open coding to identify initial concepts relevant to the research questions.

To strengthen dependability and confirmability, the two coders compared and discussed their codes to resolve discrepancies through consensus-building. New codes emerging during this process were added, and a codebook was developed to categorize and subcategorize all codes. In cases of ambiguous classification, the authors engaged in iterative discussions to achieve agreement and ensure consistency in coding logic.

The subsequent phase of qualitative theoretical analysis was carried out under the primary supervision of the first author, who provided oversight and review. The second author offered reflective feedback on the structure and content of the codebook. Both authors reached full agreement on the final themes and sub-themes, ensuring coherence and representativeness in the results.

### 3.7 Participants

This study included 12 research participants: 58% were female and 42% were male; 83% were over 65 years old and 17% were 55–64 years old; 42% had a university education, 42% had a high school education, and 16% had a junior high school education or below; 50% had some technology experience, 25% were proficient, and 25% were not proficient. Table 2 shows the study participants.

**TABLE 2:** Lists the gender, age, education level, and technology experience of the study participants.

Participant number	Gender	Age	Education level	Technology experience
A1	Female	Over 65 years	Junior high school or below	Not proficient
A2	Female	Over 65 years	High school	Some experience
A3	Male	Over 65 years	University	Some experience
A4	Male	Over 65 years	University	Some experience
A5	Male	55–64 years	University	Proficient
A6	Female	55–64 years	University	Proficient
A7	Male	Over 65 years	High school	Proficient
A8	Female	Over 65 years	High school	Some experience
A9	Male	Over 65 years	University	Not proficient
A10	Female	Over 65 years	High school	Some experience
A11	Female	Over 65 years	High school	Some experience
A12	Female	Over 65 years	Junior high school or below	Not proficient

## 4 Results

### 4.1 Technology Acceptance Model

**FIGURE 1:**
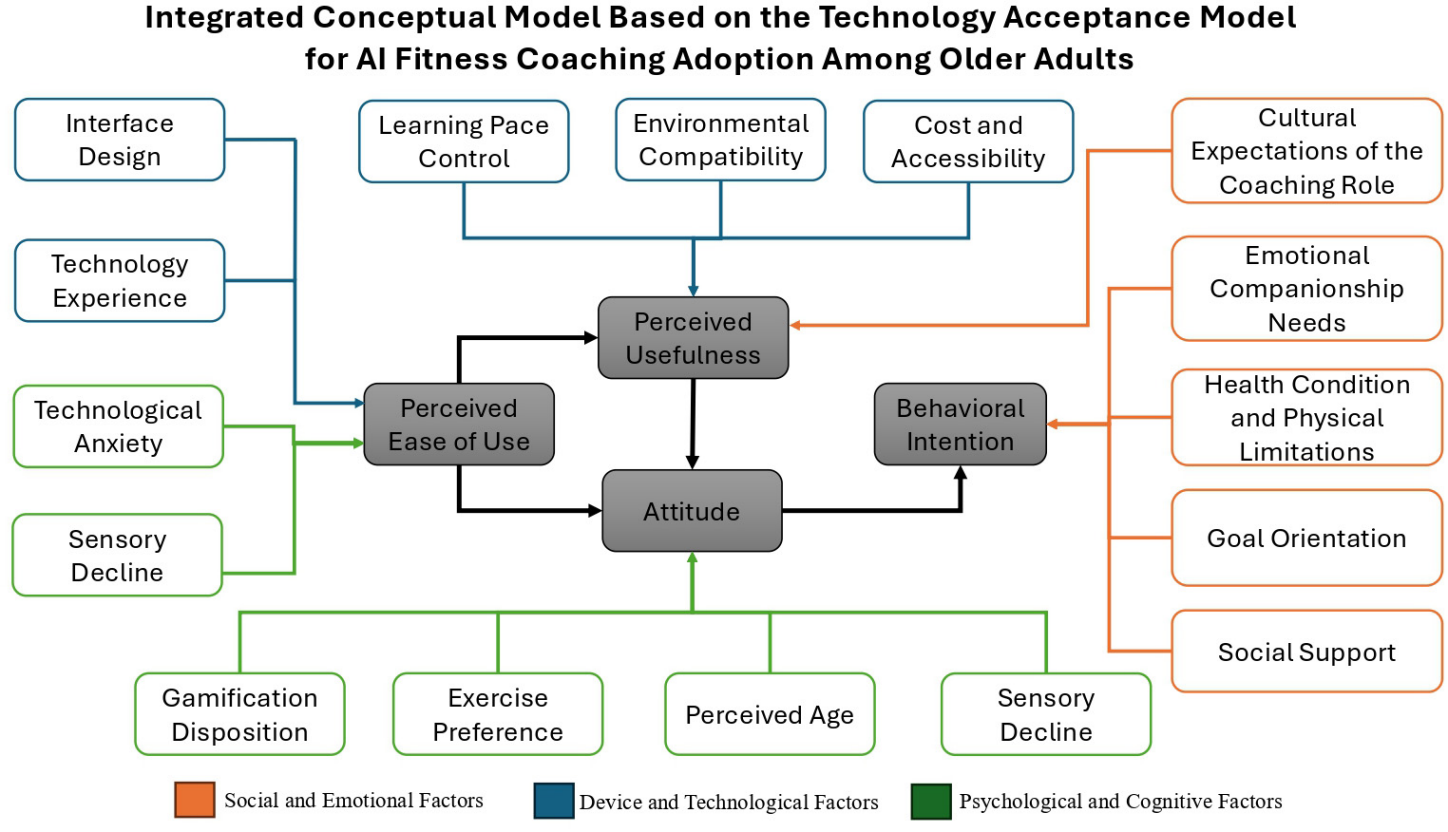
Integrated conceptual model based on the Technology Acceptance Model for AI fitness coaching adoption among older adults.

To illustrate the enablers and barriers identified across each TAM construct, Table 3 provides a synthesized matrix based on older adults’ interview responses. Figure 1 presents an integrated conceptual model grounded in the TAM, illustrating older adults’ acceptance of AI fitness coaching technologies. The model incorporates technological, psychological, and sociocultural external variables, highlighting how these dimensions interact to shape the core constructs of perceived ease of use, perceived usefulness, and user attitude, ultimately influencing behavioral intention. By reconceptualizing TAM as a dynamic and interactional framework, this model reflects the real-world complexity of aging with AI-driven health technologies, offering a more context-sensitive understanding of older adults’ adoption processes.

**TABLE 3:** Technology Acceptance Model (TAM)-based analysis of enablers and barriers.

Constructs	Enablers	Barriers constructs
Perceived ease of use	1. Intuitive operational flow and clear system logic	1. Initial instruction and guidance still needed
	2. Replay and flexible control enhance learning adaptability	2. Visual interface design misaligned with older adults’ needs
	3. Device familiarity facilitates smoother operation	
Perceived usefulness	1. Ability to assess movement	1. Lack of personalization and real-time interaction
	2. Accuracy and improve exercise quality	
	3. Enhancing self-regulation and learning ability	2. Limited value for experienced users
	4. Injury prevention and safety enhancement	
	5. Professional support in the absence of a human coach	
Attitude toward AI coaches	1. Perceived novelty and enjoyment enhancing exercise motivation	1. Emotional detachment or discomfort with non-human interaction
	2. Feelings of companionship, safety, and trust	2. Doubts about professionalism or limited effectiveness
	3. Willingness to continue using and recommend to others	

#### 4.1.1 Perceived ease of use

The analysis yielded three enabling themes and two barrier themes regarding perceived ease of use. The enablers included: (1) intuitive operational flow and clear system logic; (2) replay and flexible control enhancing learning adaptability; and (3) device familiarity facilitating smoother operation. The barriers consisted of: (1) the need for initial instruction and operational guidance; and (2) visual interface designs misaligned with the needs of older adults.

##### 4.1.1.1 Enablers

###### 4.1.1.1.1 Intuitive operational flow and clear system logic

Most participants with varying levels of technological experience emphasized that a simple, clear, and intuitive operating process was crucial in establishing a sense of comfort when first engaging with the virtual fitness coach. Several interviewees noted that well-designed buttons and step-by-step operational flows effectively reduced confusion and enhanced their sense of control. These responses suggest a general preference for consistent and predictable interface design, which aligns with a core principle of the TAM: PEOU when cognitive load during interaction is reduced.

“It’s very simple–just press the buttons and follow the steps. I think the video speed is fine; everything is clear to see.” (A2)

“I’m quite familiar with using a smartphone, so there shouldn’t be any problem with the operation. The button size and video speed are both fine.” (A4)

“Once you use it a few times, it becomes familiar. It’s not hard.” (A5)

“The operation is fine–just press and do it.” (A7)

###### 4.1.1.1.2 Replay and flexible control enhance learning adaptability

Participants (A3, A7) emphasized that the system’s “replay” and “pause” functions enabled them to review movements at their own speed, reducing frustration when unable to follow the instructor in real time. Similarly, A8 described replay as a useful adaptation to slower reaction times. Overall, the ability to pause, replay, or adjust speed enhanced learning adaptability, supported self-efficacy, and allowed for error correction without social pressure. This was an especially valuable feature for older adults with diverse physical capacities.

“If I don’t see it clearly, I can just replay it a few times–then I won’t make mistakes.” (A3)

“I can watch it again and again until I get it.” (A7)

“The video is a bit fast, so I can’t quite keep up. But I can replay it and watch slowly–that makes me feel more at ease.” (A8)

###### 4.1.1.1.3 Device familiarity facilitates smoother operation

Participants with regular experience using smartphones or tablets (A4, A5, A7, A11) reported smoother interaction with the virtual fitness coach, as familiarity with interface logic increased confidence and engagement. Connecting the system to familiar devices (e.g., TV, iPad) improved visual clarity and focus, further reducing learning barriers. In contrast, participants lacking prior 3C experience (A9) expressed hesitation, eye fatigue, and resistance. Overall, device familiarity and interface accessibility jointly lowered the learning threshold, strengthened perceived ease of use, and increased willingness to adopt the system.

“I use a smartphone, so this is no problem.” (A4)

“It’s all fine to use–I’m quite familiar with it. The speed is fine, and everything looks clear.” (A5)

“Connecting it to the TV makes it even clearer.” (A7)

“I have a smartphone and an iPad, but I usually don’t use them. I don’t like using these things–they hurt my eyes.” (A9)

“Watching it on an iPad makes the movements quite clear.” (A11)

##### 4.1.1.2 Barriers

###### 4.1.1.2.1 Initial instruction and guidance still needed

While most older adults found the virtual fitness coach simple, participants’ adaptation varied by education and technological experience. Those with higher familiarity (e.g., A5) learned through exploration, whereas less experienced users (e.g., A3, A10) relied more on instruction and expressed anxiety. Yet, even brief demonstrations helped novices (e.g., A8) adapt quickly. Overall, perceived ease of use was shaped by the interplay of cognitive ability, tech familiarity, and social support–showing that guided onboarding and repeated practice effectively transformed anxiety into confidence and sustained engagement.

“Someone needs to explain it to us first; otherwise, we don’t understand many of the buttons and can’t press them.” (A3)

“At first, I needed time to figure it out.” (A5)

“We need someone to teach us. If someone shows us how, we’ll learn. After using it once or twice, it should be fine.” (A8)

“If no one teaches me, I’m afraid of pressing the wrong thing.” (A10)

###### 4.1.1.2.2 Visual interface design misaligned with older adults’ needs

Participants reported that small screens, thin fonts, unclear buttons, and fast playback speeds hindered operation. A3 and A6 noted visibility issues linked to presbyopia, while even tech-savvy users (e.g., A7) required TV connections for clarity. Less experienced participants (e.g., A9, A11) struggled with pace and screen size, and A10 highlighted the need for clearer audio. These findings show that education and device familiarity cannot fully offset sensory decline; age-friendly design–featuring larger fonts, higher contrast, adjustable speed, and multisensory feedback–is essential to reduce visual strain and enhance perceived ease of use.

“The screen is too small, and the buttons aren’t obvious.” (A3)

“I have presbyopia–I can’t read the text clearly.” (A6)

“Only when I connect it to the TV can I see it more clearly.” (A7)

“A bit fast–I’m a bit slow,” (A9)

“The text should, of course, be large, and the sound must be clear, because many people have hearing loss.” (A10)

“It doesn’t work well on my phone.”(A11)

#### 4.1.2 Perceived usefulness

The analysis identified four enabling themes and two barrier themes related to perceived usefulness. The enablers included: (1) the ability to assess the correctness of movements, thereby enhancing exercise quality; (2) support for self-regulation and learning ability; (3) prevention of exercise-related injuries and promotion of safety; and (4) provision of professional guidance in the absence of a human coach. The barriers included: (1) lack of personalization and real-time interaction, leading to limited effectiveness; and (2) perceived limited benefits for individuals with prior exercise experience.

##### 4.1.2.1 Enablers

###### 4.1.2.1.1 Ability to assess movement accuracy and improve exercise quality

Participants across ages and technology backgrounds agreed that movement monitoring and instant feedback helped them identify errors and improve performance. Even less tech-savvy users (A1, A2) appreciated that “the system corrects mistakes” and “prevents doing it wrong,” while more experienced users (A3, A4, A5, A7) highlighted its role in judging accuracy and enhancing effectiveness. Overall, real-time corrective feedback not only improved exercise quality and confidence but also motivated continued engagement, serving as a key driver of perceived usefulness and sustained adoption.

“Because it responds, I won’t do it wrong.” (A1)

“When we do it by ourselves, we don’t even know what we’re doing wrong.” “It corrects the wrong parts, so I know if I’m doing it right.” (A2)

“It judges right or wrong–I can refer to it.” (A3)

“If it can show me I did it wrong, of course that’s important.” (A4)

“Help us perform the movements correctly in order to achieve results.” (A5)

“It can correct the parts we did wrong.” (A7)

###### 4.1.2.1.2 Enhancing self-regulation and learning ability

Participants across age and education levels agreed that real-time feedback and monitoring enabled independent learning and movement correction. For less experienced users (A1), the AI coach offered companionship and motivation to sustain routines, while educated or tech-savvy participants (A3, A5, A6) valued its feedback reports and error alerts for refining movements–partially substituting for human guidance. Overall, the system’s visual and feedback functions strengthened users’ self-monitoring and continuous improvement, promoting autonomy, confidence, and lifelong learning.

“With it guiding me, I will keep moving. Otherwise, if I’m alone, I’ll lose motivation after a while.” (A1)

“I can correct myself–just try a few more times.” (A3)

“It gives a report, so I know what I did wrong and can do it again.” (A5)

“The monitoring system can tell me what I’m doing right and wrong,” (A6)

“The report shows me how to adjust, so I’ll pay attention next time.” (A11)

###### 4.1.2.1.3 Injury prevention and safety enhancement

Participants widely valued the virtual fitness coach’s real-time monitoring and feedback as safeguards against exercise errors and injuries. Less tech-savvy users (e.g., A2) appreciated its objective, consistent guidance, while experienced users (A4, A5) described a heightened “sense of safety” and “behavioral trust.” Others (A8, A11) highlighted preventive reminders that “help avoid injuries.” Overall, safety assurance enhanced users’ trust, control, and self-efficacy, forming a critical psychological link between perceived safety and sustained engagement with AI exercise systems.

“What friends say isn’t always correct, but the monitoring system can correct mistakes,” “If I know what’s right or wrong, it hurts less.” (A2)

“It reminds me if I do it wrong–that’s safer.” (A4)

“It makes me less afraid of getting injured while exercising.” (A5)

“If I don’t do it wrong, I won’t get injured.” (A8)

“It gives reminders in advance, which can prevent many exercise injuries.” (A11)

###### 4.1.2.1.4 Professional support in the absence of a human coach

Across backgrounds, participants viewed the virtual fitness coach as providing consistent, real-time, and reliable guidance that effectively substituted for human instructors. Even less experienced users (A1, A8) described it as “more professional than a regular video” and “like having a teacher beside me,” while educated and tech-savvy users (A4–A6) valued its consistent quality and instant access to knowledge. Overall, the system’s instructional credibility and feedback precision positioned it as a dependable self-learning tool that bridges the gap between human coaching and autonomous exercise.

“It’s impressive–more professional than a regular video.” (A1)

“Even though it’s a machine, it feels like a coach.” (A4)

“The virtual coach delivers consistent quality, unaffected by differences among human coaches.” (A5)

“The virtual coach allows me to quickly access the knowledge I need.” (A6)

“It corrects me–just like having a teacher next to me.” (A8)

##### 4.1.2.2 Barriers

###### 4.1.2.2.1 Lack of personalization and real-time interaction

Participants widely reported that the virtual fitness coach could not adjust to individual needs or provide responsive feedback. Even educated and tech-savvy users (A3, A6) noted it “does not respond to questions,” while less experienced users (A9) criticized its repetitive content. Moderately experienced participants (A10, A11) further pointed out its lack of emotional connection and sensitivity to subtle movements. Overall, the system’s limited adaptability and social responsiveness hindered long-term engagement and failed to replicate the individualized support of human coaching.

“The virtual coach is fixed–if you don’t understand something, there’s no response,” (A3)

“It’s not like a coach who knows what I can’t do–it just says what it says.” (A6)

“It says the same things every time–no variation.” (A9)

“Friends or coaches care about me; the virtual one doesn’t give that feeling,” “The movements are all the same–it doesn’t adjust to me.” (A10)

“Small techniques can’t be seen just by watching,” (A11)

###### 4.1.2.2.2 Limited value for experienced users

Older adults with higher education or extensive exercise backgrounds found the virtual coach’s basic, repetitive content insufficient for their advanced needs. Even motivated, tech-competent users (A3, A6) described it as offering only simple “reminders,” while A10 remarked she might be “better than the coach.” Without adaptive difficulty or deeper guidance, the system risks losing credibility and engagement among skilled older users.

“It just reminds me–I don’t think it makes much of a difference.” (A3)

“I’ve been exercising for a long time–if I already know everything it offers, then of course it’s not very helpful.” (A6)

“I might not be any worse than the virtual coach–maybe I could even critique it.” (A10)

#### 4.1.3 Attitude toward AI fitness coaches

The analysis identified three enabling themes and two barrier themes regarding users’ attitudes toward AI fitness coaches. The enablers included: (1) a sense of novelty and enjoyment, which enhanced motivation to exercise; (2) feelings of companionship, safety, and trust; and (3) willingness to continue using and recommend the system. The barriers included: (1) emotional detachment from technology or discomfort with non-human interaction; and (2) doubts about the system’s professionalism or limited perceived effectiveness.

##### 4.1.3.1 Enablers

###### 4.1.3.1.1 Perceived novelty and enjoyment enhancing exercise motivation

Across education and technology levels, participants found the system’s interactive and game-like features stimulating, increasing curiosity and willingness to exercise. Less experienced users (A1) described it as “interesting and new,” while A3 likened it to “playing a video game” that encouraged persistence. More tech-familiar users (A5, A7) noted its “enjoyment” and “confidence-boosting” effects, and A11 highlighted how encouragement fostered curiosity and self-efficacy. Overall, the system’s novelty, feedback, and playful challenge effectively triggered intrinsic motivation, particularly among older adults seeking new or independent exercise experiences.

“I think it’s quite interesting–I’ve never tried something like this before.” (A1)

“The encouragement is like playing a video game–when you pass a level, you move on; when you fail, you keep trying. It really motivates me to exercise.” (A3)

“When I follow it, I feel it’s more interesting and gives me a bit more confidence.” (A5)

“I feel that new technologies like this are worth trying out.” (A7)

“With encouragement, I feel curious and want to see if I can do better next time.” (A11)

###### 4.1.3.1.2 Feelings of companionship, safety, and trust

Participants widely described the virtual fitness coach as providing reassurance and a sense of presence during exercise. Less experienced users (A1, A2) felt “accompanied” and more motivated, while educated users (A4, A8) emphasized that real-time correction made them “feel safer.” A7 noted it was “like exercising with someone,” showing the system’s potential to meet emotional companionship needs. Overall, the coach’s feedback and presence fostered trust, safety, and emotional support–key drivers for sustained engagement among older adults.

“When it guides me, I keep moving; otherwise, when I’m alone, I stop exercising after a while.” (A1)

“It’s like someone is next to me teaching–I feel more at ease.” (A2)

“It reminds me when I do it wrong, so I feel more secure.” (A4)

“It feels like having someone exercise with you – the virtual coach is great.” (A7)

“It corrects my movements, so I feel safer.” (A8)

“It’s like having someone right there accompanying me.” (A10)

###### 4.1.3.1.3 Willingness to continue using and recommend to others

Participants across education and technology levels expressed readiness to keep using and share the AI fitness coach with others, reflecting both trust and integration into daily routines. Less experienced users (A1, A2) valued its flexibility and accessibility, while educated users (A4, A7, A11) highlighted ease of use and intention to recommend it to family. Overall, the system’s convenience, reliability, and user-friendliness supported sustained engagement and social diffusion among older adults.

“I would use it quite often. Whenever I have time, I can move and exercise.” (A1)

“If it’s available, I’ll follow along and learn from it.” (A2)

“If possible, I’d recommend it to my family.” (A4)

“If I can use it again, I’d like to continue.” (A7)

“If it’s easy to use, I think many people would be willing to try it.” (A11)

##### 4.1.3.2 Barriers

###### 4.1.3.2.1 Emotional detachment or discomfort with non-human interaction

Many participants noted that while AI fitness coaches provided functional value, they lacked the warmth and empathy of human interaction. Users (A6, A8, A9) described the system as “robotic,” “cold,” and lacking genuine encouragement, reflecting discomfort with non-human communication. Without humanized feedback or contextual interaction, the system risks emotional detachment, reducing affective acceptance and long-term use among older adults.

“I still prefer a real coach–there’s interaction.” (A6)

“It just keeps talking–it feels robotic.” (A8)

“When there are other classmates on-site, the atmosphere becomes lively. It’s not like using a 3C product, where no one really cares,” “It’s not like a person who encourages you–it feels cold.” (A9)

###### 4.1.3.2.2 Doubts about professionalism or limited effectiveness

Experienced older adults questioned the system’s depth and credibility, noting that repetitive and basic content offered little value beyond existing knowledge. Participants (A3, A6, A10) felt the coach “didn’t help much” or “made no difference,” reflecting unmet expectations for advanced instruction and personalization. Without adaptive feedback or professional-level guidance, the system risks appearing redundant and failing to sustain engagement among skilled users.

“Many apps are designed like this, so it doesn’t really help much,” “It doesn’t feel as effective as a real class.” (A3)

“I’ve been exercising for a long time. If I already know everything it teaches, of course it’s not very useful.” (A6)

“I can do these movements on my own–it makes no difference.” (A10)

### 4.2 Emerging external variables

#### 4.2.1 Device and technological factors

This section identifies five emerging external variables under the category of device and technological factors: Interface and Voice Design Sensitivity, Technology Usage Experience, Environmental Fit, Cost and Accessibility, and Learning Pace Control. As illustrated in Table 4, these variables are supported by corresponding excerpts and thematic analysis.

**TABLE 4:** Device and technological factors.

Emerging variable	Representative quotes (participant ID)	Thematic interpretation
Interface and voice design sensitivity	“The voice sounds like a machine–very monotonous.” (A9) “The interface is stiff and emotionless.” (A6)	Unnatural visual and auditory design elements may evoke emotional coldness and rejection, reducing both attitude toward use and perceived ease of use.
Technology usage experience	“I regularly use smartphones and tablets.” (A11) “I’m not very good with tech.” (A6)	Prior experience with digital devices influences familiarity and trust, serving as a foundational condition for perceived ease of use.
Environmental fit	“My living room is too small–I can’t stretch out.” (A7) “There are too many people at home–I feel embarrassed using it.” (A2)	Limited space and lack of privacy can hinder the feasibility of using AI fitness systems in home settings.
Cost and accessibility	“If it’s expensive, I won’t buy it.” (A6) “Where can I use it? Can I set it up at home?” (A10)	Financial constraints and limited access to equipment are external barriers to actual adoption.
Learning pace control	“If it’s too fast, I can’t keep up–it should go slower.” (A5) “It would be better if I could control the pace myself.” (A8)	Older adults have diverse learning speeds; the ability to control pace directly affects motivation and willingness to continue using the system.

#### 4.2.2 Psychological and cognitive factors

This section identifies six emerging external variables related to psychological and cognitive dimensions: Self-efficacy, Technology Anxiety, Perceived Age, Sensory Decline, Gamification Disposition, and Exercise Preference. As shown in Table 5, these variables are illustrated through representative quotes and thematic interpretations.

**TABLE 5:** Psychological and cognitive factors.

Emerging variable	Representative quotes (participant ID)	Thematic interpretation
Self-efficacy	“I watch videos–so I can learn this too.” (A4) “I can adjust my posture by myself.” (A3)	Users expressed confidence in their ability to operate the system and make self-corrections, which positively influenced their acceptance and attitude toward the AI coach.
Technology anxiety	“I’m afraid of pressing the wrong button–what if it breaks?” (A10) “Still scared of computers–not sure where I’m clicking.” (A6)	Fear of making mistakes or damaging the device created initial resistance among some older adults, reducing perceived ease of use.
Perceived age	“I can still learn–I’m not that old yet.” (A8) “This is for young people–I don’t think it suits me.” (A10)	One’s self-perception of age and learning capability influenced their intention and confidence to use the system, acting as a psychological external factor.
Sensory decline	“The text is too small–I can’t read it clearly.” (A6) “It sounds too robotic.” (A9)	Visual and auditory impairments affected interface usability and message comprehension, thus weakening perceived ease of use.
Gamification disposition	“That kind of encouragement feels like a video game… I get competitive if I don’t succeed.” (A3)	Participants who responded well to gamified features (e.g., points, challenges) showed greater motivation for continued engagement.
Exercise preference	“I prefer walking–this is not what I had in mind.” (A3) “These moves are too fast–I’m used to slower ones.” (A9)	A mismatch between the system’s design and users’ preferred exercise style led to discomfort and potential rejection.

#### 4.2.3 Social and emotional factors

This section identifies five emerging external variables pertaining to social and emotional dimensions: Social Support, Cultural Expectation of Coaching Role, Health Condition and Physical Limitation, Goal Orientation, and Emotional Companionship Need. Table 6 presents the representative participant quotes and thematic interpretations for each variable.

**TABLE 6:** Social and emotional factors.

Emerging variable	Representative quotes (participant ID)	Thematic interpretation
Social support	“My friend said it was good, so I gave it a try.” (A2) “My daughter said it’s safer this way.” (A7)	Encouragement and recommendations from family and friends reduce psychological barriers to first-time use, serving as a key social influence on technology adoption.
Cultural expectation of coaching role	“I still prefer a real person–it feels more authentic.” (A6) “Talking to a person feels more natural–machines lack warmth.” (A9)	Older adults often hold culturally embedded expectations of the coach as a human figure, leading to reservations toward AI-based coaching.
Health condition and physical limitation	“Sometimes my knees hurt–I can’t do it.” (A9) “My shoulder isn’t that good–it doesn’t know that.” (A6)	Physical limitations reduce adaptability to standardized AI instructions and erode trust in its feedback, thus weakening perceived usefulness.
Goal orientation	“I just want something simple–not too complicated.” (A3) “If it could help me lose weight, that would be even better.” (A1)	When the system aligns with users’ personal health or appearance-related goals, it increases perceived value and motivation to use.
Emotional companionship need	“It feels like someone is watching me do it.” (A4) “Without a real person, it feels a bit lonely.” (A9)	If AI lacks interactivity and companionship features, it fails to fulfill older adults’ emotional needs, limiting long-term engagement.

## 5 Discussion

### 5.1 Applicability and limitations of TAM in the context of older adults

This study partially supports the applicability of the TAM to older adults’ use of AI fitness coaching systems, particularly in relation to the two core constructs: PEOU and PU. Most participants found the system intuitive, with a clear process and user-friendly interface, which reduced the learning threshold (Section “4.1.1.1.1 Intuitive operational flow and clear system logic”). These findings are consistent with Davis (1989) and Dakulagi et al. (2025), who emphasized that interface design plays a critical role in enhancing technology adoption intentions. In addition, Younas et al. (2025) highlighted that technological proficiency, institutional support, and psychological adaptability are decisive factors shaping successful technology adoption. This perspective resonates strongly with the multifaceted challenges that older adults encounter in developing ease of use and sustaining engagement.

However, the formation of PEOU among older adults is not solely determined by design characteristics. Physiological limitations (e.g., visual decline), psychological barriers (e.g., fear of operation), and cognitive load (e.g., memory burden) can all diminish perceptions of ease of use. For instance, despite simplified designs, some participants still expressed concerns such as “afraid of pressing the wrong button” or “don’t know where to start,” (Section “4.1.1.2.1 Initial instruction and guidance still needed”) indicating that their perception of “ease” is mediated by sensory abilities and perceived learning costs (Czaja, 2019). Thus, the rational pathway assumed by the original TAM regarding the formation of PEOU appears overly simplistic in the context of aging populations.

Based on these findings, this study suggests that the construct of PEOU in the TAM should be expanded to incorporate “learning supportiveness” and “sensory adaptability” as external variables. Older adults’ perceived ease of use is dynamically developed through repeated practice, visual guidance, and feedback correction rather than through a single exposure. Accordingly, future AI fitness systems should adopt “progressive onboarding” and “multi-sensory interface design” principles–such as adjustable playback speed, larger fonts, and voice-guided tutorials–to facilitate confidence and reduce cognitive anxiety during initial engagement.

Similarly, disparities were observed in perceptions of PU. For older adults with limited exercise experience or those unable to attend in-person classes regularly, AI fitness coaches provided real-time feedback and guidance that were seen as practical and valuable alternatives (Sections “4.1.2.1.1 Ability to assess movement accuracy and improve exercise quality,” “4.1.2.1.2 Enhancing self-regulation and learning ability,” “4.1.2.1.3 Injury prevention and safety enhancement”). Participants described the system as being able to “correct wrong movements” (Section “4.1.2.1.1 Ability to assess movement accuracy and improve exercise quality”), “help us do it right and get results” (Section “4.1.2.1.1 Ability to assess movement accuracy and improve exercise quality”), and “give reminders to prevent injuries” (Section “4.1.2.1.3 Injury prevention and safety enhancement”). Conversely, individuals with prior training experience or high self-efficacy found these features redundant and even disruptive to their learning rhythm (Section “4.1.2.2.2 Limited value for experienced users”). For example, they noted that the system “repeats what I already know” or “doesn’t provide challenge or deeper guidance” (Section “4.1.2.2.2 Limited value for experienced users”). This suggests that as users’ competencies increase, the system’s perceived added value may diminish, illustrating a marginal utility effect. In other words, when users already possess sufficient knowledge and skills, the system’s basic functions no longer offer added value; instead, repetitive content and lack of challenge may undermine their motivation for continued use. This finding challenges the linear assumption in prior studies that higher self-efficacy leads to higher PU (An et al., 2024). In contexts involving physical execution and motor judgment, PU should not be treated as a static assessment but rather as a dynamic, individualized, and context-dependent perception.

Moreover, Menhas et al. (2024) found that nature-based social prescriptions enhance mental health by fostering psychological restoration, emotional regulation, and social connectedness. Similarly, AI-based fitness coaching can evoke restorative experiences by promoting autonomy, perceived control, and positive affect. These parallels suggest that perceived usefulness should encompass not only functional outcomes but also emotional and restorative dimensions in older adults’ technology acceptance.

From a theoretical standpoint, this indicates that PU is subject to a “marginal utility effect” wherein perceived usefulness decreases with user proficiency. Future extensions of TAM in the aging context should therefore conceptualize PU as a dynamic construct, moderated by factors such as self-efficacy, physical literacy, and perceived challenge. From a design perspective, systems should incorporate adaptive difficulty and tiered content to maintain continuous engagement across varying ability levels. Moreover, policy makers promoting AI-based exercise systems in senior care centers should establish functional grading standards to match system complexity with user capability, thereby ensuring equitable access and sustained motivation.

Moreover, attitudes toward the system also diverged significantly (Section “4.1.3 Attitude toward AI fitness coaches”). While some participants demonstrated positive attitudes due to novelty and curiosity (Section “4.1.3.1.1 Perceived novelty and enjoyment enhancing exercise motivation”), others expressed emotional disconnect and distrust (Section “4.1.3.2.1 Emotional detachment or discomfort with non-human interaction”), citing the lack of “human warmth” and the “robotic tone” of the system. Participants described it as “fun like a game” (Section “4.1.3.1.1 Perceived novelty and enjoyment enhancing exercise motivation”) or “like having a coach with me” (Section “4.1.3.1.2 Feelings of companionship, safety, and trust”), but others said “it feels cold” or “not like a person who encourages you” (Section “4.1.3.2.1 Emotional detachment or discomfort with non-human interaction”). This suggests that older adults’ acceptance of technology is not solely based on rational assessments of utility but is also influenced by emotional compatibility and interaction quality (Dosso et al., 2022). Although previous research posited that older adults’ acceptance is primarily shaped by pragmatic trust, with affective trust playing a minor role (Zafrani, 2022), our findings reveal that even if a system is perceived as functionally effective, a lack of emotional warmth may still lead to resistance (Sections “4.1.3.2.1 Emotional detachment or discomfort with non-human interaction,” “4.1.3.2.2 Doubts about professionalism or limited effectiveness”). These results highlight the necessity of extending TAM to include emotional and social dimensions–specifically, “affective attitude” and “social presence.” Designers should embed affective computing elements such as personalized encouragement, human-like voice tones, and empathic feedback to strengthen perceived companionship and trust. On a policy level, government and institutional procurement guidelines should consider emotional engagement metrics (e.g., perceived warmth, trust, and social support) as key indicators when evaluating senior-oriented AI health technologies.

### 5.2 Contradictory experiences within TAM constructs

To further understand older adults’ cognitive and emotional responses when engaging with AI fitness coaching systems, this study analyzed the contradictions embedded in their acceptance experiences through the three core TAM constructs. The findings reveal that even when participants expressed functional approval, they often simultaneously experienced both enabling and inhibiting psychological states during system use. This highlights that technology acceptance is a context-dependent, non-linear, and continuously evolving process.

#### 5.2.1 Perceived ease of use

Some participants described the system as logically structured, with replay and flexible control features that supported autonomy in learning (Sections “4.1.1.1.1 Intuitive operational flow and clear system logic,” “4.1.1.1.2 Replay and flexible control enhance learning adaptability”). This aligns with Liu et al. (2022), who emphasized that technologies designed for older adults should enhance engagement and user agency rather than act merely as functional replacements. For example, A3 stated, “If I don’t see it clearly, I can just replay it a few times, then I won’t make mistakes” (Section “4.1.1.1.2 Replay and flexible control enhance learning adaptability”).

However, several participants reported feeling nervous, afraid of making mistakes, or uncertain about whether they were using the system correctly during their first interaction (Section “4.1.1.2.1 Initial instruction and guidance still needed”). This indicates that even a well-designed interface cannot easily eliminate the psychological defensiveness and unfamiliarity experienced in the early stages of use (Tajudeen et al., 2022). A10 noted, “If no one teaches me, I’m afraid of pressing the wrong thing” (Section “4.1.1.2.1 Initial instruction and guidance still needed”). This pattern of initial anxiety followed by gradual adaptation suggests that PEOU is shaped by temporal and situational factors and should be understood as a dynamic perception rather than a static attribute.

As participants became more familiar with the system, several developed personalized strategies for navigating and using its features. For instance, A8 shared, “At first I had to watch everything many times, but later I figured out how to skip or select the parts I needed.” This kind of exploratory use reflects a gradual shift from passive learning to active control.

Research on technology learning among older adults supports this pattern. As older users engage in repeated trials and experimentation, their initial uncertainty about the system tends to decline, which in turn enhances their perception of usability (Barnard et al., 2013). Over time, frequent engagement not only reduces ambiguity but also fosters skill mastery and confidence. As Kebede et al. (2022) observed, continued interaction with technology can lead many older adults to become competent and self-assured users.

According to research, the challenges older adults face when using technology stem not only from physiological or sensory limitations but also from age-related declines in cognitive functions such as working memory, attention, and information processing speed. Even when user interfaces are simplified, these cognitive factors can still create barriers to effective use. In practice, only through sufficient instruction, repeated practice opportunities, and guided assistance can anxiety and frustration be reduced, thereby enhancing perceived usability and willingness to use the technology (Czaja, 2019; Kamin et al., 2020).

In addition, social support, including assistance and companionship from peers, family members, or instructors, has been shown to facilitate older adults’ adoption and sustained use of new technologies (Kamin et al., 2020). For example, A5 stated, “When the teacher or classmates are beside me, I feel more confident using it,” clearly illustrating how social interaction positively contributes to confidence in system operation. Therefore, in the design and implementation of fitness technologies or AI fitness coaching systems, it is essential to consider these external support factors in order to foster learning and acceptance among older users.

#### 5.2.2 Perceived usefulness

A similarly dual pattern emerged regarding PU. On one hand, many participants appreciated the system’s real-time feedback and posture correction features, which supported exercise safety and self-monitoring. These experiences demonstrated the system’s professional and functional value (Sections “4.1.2.1.1 Ability to assess movement accuracy and improve exercise quality,” “4.1.2.1.3 Injury prevention and safety enhancement,” “4.1.2.1.4 Professional support in the absence of a human coach”). For instance, A2 commented, “It corrects the wrong parts, so I know if I’m doing it right” (Section “4.1.2.1.1 Ability to assess movement accuracy and improve exercise quality”).

On the other hand, some users perceived the responses as overly standardized and impersonal. One participant even expressed discomfort at “feeling monitored,” which led to a sense of rejection (Section “4.1.2.2.1 Lack of personalization and real-time interaction”). A10 remarked, “The movements are all the same, it doesn’t adjust to me,” highlighting a lack of personalized responsiveness. These contradictory responses suggest that PU is influenced by users’ perceptions of bodily autonomy, self-competence, and prior experiences.

Previous research has shown that older adults’ emphasis on autonomy and decision-making is deeply rooted in their accumulated life experiences, cultural background, and personal identity (Gilleard, 2022). This implies that even when a system is objectively useful, its failure to convey respect for the user’s bodily context and individuality may hinder the formation of PU. Therefore, PU should not be regarded solely as a functional evaluation but should be understood as the result of interactions between bodily self-perception and subjective engagement.

To better understand this complexity, it is important to consider the influence of sociocultural factors on PU. Technology acceptance theories developed in Western contexts often prioritize individualistic, rational, and autonomy-based decision-making. However, empirical studies in Chinese-speaking regions have revealed distinctive patterns of risk sensitivity, institutional trust, and intergenerational decision-making that shape older adults’ attitudes toward AI-driven health systems. For example, older adults in Taiwan and mainland China often prioritize tangible risks such as financial burden or technical malfunction over more abstract concerns such as data privacy or emotional discomfort (Yang et al., 2023; Huang T. et al., 2024). This emphasis on concrete risk reflects both limited digital literacy and a cultural preference for social stability and institutional reliance (Chen and Cheng, 2022). Furthermore, trust in AI technologies is often based on previous service experiences and interpersonal interactions, rather than institutional guarantees (Fu and Ma, 2025).

In the Chinese cultural context, technology adoption is not simply an individual decision. It is frequently a negotiated outcome shaped by family dynamics. The concept of collaborative adoption, as proposed by Jin and Qu (2025), describes how children or family caregivers often act as mediators and proxy decision-makers. They guide, justify, and sometimes even execute decisions to adopt or reject AI systems. In healthcare-related contexts, where moral responsibility and perceived risk are heightened, older adults often delegate such decisions to trusted family members (Ye et al., 2025). In these cases, PU becomes closely tied to the caregiver’s trust, emotional assurance, and willingness to share responsibility.

These findings challenge the individual-centered assumptions of traditional TAM and call for a reconceptualization of PU as a construct embedded in social and cultural contexts. In collectivist societies, where family members frequently serve as decision-making agents and emotional mediators, PU emerges through interpersonal negotiation, relational trust, and shared meaning-making. It should therefore be viewed as a situated and relational perception that varies depending on the context of use, generational roles, and perceived accountability for risk. This perspective supports the development of AI systems that are sensitive to the broader social environments of older users, rather than focusing only on one-to-one user interaction with the system.

Additionally, this study observed a marginal utility effect in PU, especially among experienced users with high self-efficacy. Participants such as A6 and A10 noted that the system “repeats what I already know” or “makes no difference,” suggesting that once a user’s skill level surpasses the system’s instructional capacity, the perceived usefulness may stagnate or decline. This finding contradicts the classical TAM assumption that PU increases proportionally with user competence. We therefore propose that PU should be treated as a dynamic and conditional construct, where its sustainability depends on a balanced interaction between functional value, novelty, and level of challenge. Designing adaptive content and progressive task structures may help maintain perceived usefulness across varying user abilities.

#### 5.2.3 Attitude toward use

The findings also illustrate a typical tension between cognitive acceptance and emotional resistance. While some participants recognized the AI coach’s instructional value (Sections “4.1.3.1.2 Feelings of companionship, safety, and trust,” “4.1.3.1.3 Willingness to continue using and recommend to others”), they also expressed discomfort with its “cold and mechanical voice” and “lack of interactive flexibility,” which led to emotional detachment and rejection (Sections “4.1.3.2.1 Emotional detachment or discomfort with non-human interaction,” “4.1.3.2.2 Doubts about professionalism or limited effectiveness”). A9 said, “It’s not like a person who encourages you. It feels cold” (Section “4.1.3.2.1 Emotional detachment or discomfort with non-human interaction”).

This highlights the importance of emotional connectedness (Peine and Neven, 2019) and interactional warmth (Bickmore and Picard, 2005) in shaping user attitudes. It also suggests that older adults’ acceptance of technology is influenced not only by rational evaluation but also by the system’s ability to foster psychological identification and to serve as a credible alternative to human interaction.

In response to the contradictions observed within TAM, this study proposes a triadic interaction model that includes perceived usefulness, social interaction expectation, and feedback type as three key and interrelated factors that shape user attitudes. Although older adults acknowledged the system’s functional value, many reported negative or ambivalent feelings when the feedback appeared robotic, repetitive, or socially disengaged. This indicates that perceived usefulness alone is not sufficient to ensure acceptance. Long-term engagement depends on whether the interaction feels emotionally supportive and socially responsive.

This study therefore extends the Technology Acceptance Model by emphasizing the importance of interaction design and emotional resonance in connecting perceived usefulness with user attitudes. Future research should explore how personalized feedback, empathetic tone, and adaptive timing influence older users’ perceptions of social presence and trust. These insights can help explain how emotional connection, in addition to functional utility, contributes to long-term technology acceptance among aging populations.

### 5.3 Emerging external variables and the potential for extending the TAM framework

Through qualitative analysis, this study identified 16 emerging external variables not included in the original TAM framework. These variables span three major domains: the technological environment, psychological and cognitive factors, and social and emotional influences. This highlights that technology acceptance among older adults is a highly contextualized decision-making process. While this study did not aim to validate a revised model, the findings offer a conceptual foundation for future theoretical extensions of TAM and can guide quantitative testing and model refinement in aging-related technology research.

#### 5.3.1 Technological and device-related factors

Five external variables were identified in this domain: interface and voice design sensitivity, technology usage experience, environmental fit, cost and accessibility, and learning pace control, as shown in Table 4 (Section “4.2.1 Device and technological factors”). First, unnatural visual or auditory design elements were often perceived as lacking human warmth, resulting in emotional detachment and rejection. This supports previous findings that a lack of social presence in interface design can lower older adults’ motivation and acceptance (Bickmore and Picard, 2005). From a theoretical perspective, this highlights the importance of incorporating affective computing and social presence theory into future models of AI interaction design. Integrating emotional cues such as empathetic tone, adaptive feedback, or anthropomorphic expressions could enhance the perceived relational quality of virtual coaches and strengthen perceived ease of use.

Second, participants’ familiarity with smart device operation was shown to significantly influence their trust in and perceived ease of new technology (Mitzner et al., 2010). This suggests that technology acceptance among older adults may be mediated by digital self-efficacy and prior experience, implying the need to expand the TAM framework to include “technological familiarity” as an external variable. Future studies could employ longitudinal tracking to observe how repeated exposure and guided training alter users’ perceived ease of use and trust trajectories.

Third, environmental and spatial factors were particularly salient. Participants noted that small living spaces and a lack of privacy limited the feasibility of home-based use, emphasizing the importance of household context in the design of aging-in-place technologies (Peek et al., 2014). Practically, system developers should consider modular or compact devices that accommodate space constraints, as well as adjustable camera angles and privacy protection mechanisms. Policymakers could also promote subsidies for adaptive home environments or provide community-based digital exercise zones to reduce spatial inequities among older users.

Cost and accessibility were also crucial; participants expressed concern over financial affordability and equipment installation, echoing the literature on the digital divide and cost as a barrier to adoption (Lee and Coughlin, 2015). This finding underscores the policy relevance of economic accessibility. Public health agencies and local governments could implement tiered pricing schemes or digital inclusion programs to ensure equitable access to AI fitness technologies. Theoretically, perceived economic cost could be modeled as a moderating factor that influences the relationship between perceived usefulness and behavioral intention among older adults.

Finally, older adults strongly emphasized the need for flexible control over learning pace, such as the ability to replay or adjust speed. This finding aligns with Pang et al. (2021), who noted that time flexibility and autonomy in learning are vital for older users adopting new technologies. From a design standpoint, adaptive learning systems that automatically calibrate exercise intensity and instruction speed based on user progress could enhance sustained engagement. Future research could explore how learning pace control interacts with autonomy-supportive features from Self-Determination Theory (SDT), providing a more comprehensive framework for older adults’ engagement and satisfaction.

#### 5.3.2 Psychological and cognitive factors

Six psychological and cognitive variables were identified: self-efficacy, technology anxiety, perceived age, sensory decline, gamification disposition, and exercise preference, as shown in Table 5 (Section “4.2.2 Psychological and cognitive factors”). Self-efficacy emerged as a key psychological resource positively associated with system acceptance. Previous studies suggest that higher self-efficacy leads to greater technology use and more proactive engagement with problem-solving (Pejić Bach et al., 2023), whereas lower self-efficacy may cause anxiety, avoidance, and reliance on others (Luo et al., 2025). These findings suggest that enhancing older adults’ confidence in using technology can help reduce operational anxiety and improve acceptance. In practice, the system could include step-by-step tutorials and visual feedback functions to help users gradually build a sense of mastery and safety. Consistent with Afzaal et al. (2025), intrinsic motivation serves as a psychological catalyst that transforms perceived ease of use and usefulness into active engagement, reinforcing the mediating role of self-efficacy in sustaining technology adoption.

Moreover, Younas et al. (2023) demonstrated that coping behavior significantly mediated the negative impact of pandemic stressors on physical, psychological health, and overall well-being. Their findings suggest that adaptive coping strategies, such as emotional regulation, maintaining regular physical activity, and cultivating a sense of control, serve as psychological protective mechanisms during stressful situations. In line with this perspective, the AI-based virtual fitness coach can similarly enhance perceived usefulness and ease of use while simultaneously promoting positive emotions, autonomy, and perceived control, thereby activating users’ psychological restoration processes.

Perceived age, or one’s subjective assessment of aging, also influenced usage intentions and engagement (Hu et al., 2024). This indicates that technology adoption is not solely shaped by chronological age but also by self-perceived aging. Design strategies that employ friendly language, adaptive display modes, and flexible content presentation may help reduce psychological resistance and foster a sense of familiarity and engagement.

Declining sensory abilities (e.g., poor vision, hearing loss) posed direct challenges to interface and voice design, as insufficient adaptation could reduce perceived usability (Czaja, 2019). Therefore, interfaces should incorporate multimodal feedback such as enlarged fonts, audio prompts, or high-contrast layouts to accommodate diverse sensory needs. At the policy level, developing age-friendly design guidelines could help ensure accessibility for older populations.

Participants also differed in their gamification disposition–those who enjoyed elements such as scoring or level progression reported higher motivation (Yin et al., 2022). This suggests that gamification features can motivate some users but may have limited effects for others. Future research could examine the optimal level and type of gamification for different age groups to balance motivation and cognitive load.

Finally, exercise preference had a significant impact on perceived system compatibility. When the training content aligned with users’ preferred exercise styles, enjoyment and willingness to continue increased; in contrast, mismatched content led to rejection responses (Creighton et al., 2022). This highlights the importance of offering diverse and customizable exercise content. Practically, collecting users’ preferences through questionnaires or initial setup processes could help provide personalized exercise recommendations, thereby enhancing satisfaction and long-term engagement.

#### 5.3.3 Social and emotional factors

Five additional variables emerged under the social and emotional domain: social support, cultural expectations of coaching, health condition and physical limitations, goal orientation, and emotional companionship need, as shown in Table 6 (Section “4.2.3 Social and emotional factors”). Family and peer encouragement served as strong facilitators of initial trial and usage, consistent with the role of subjective norms and social influence in TAM extensions (Venkatesh and Davis, 2000). This finding indicates that technology adoption among older adults is often socially mediated rather than purely individual. In practical terms, integrating family participation or community-based programs into AI fitness interventions could reinforce motivation and reduce abandonment rates. Future studies could further explore how social influence interacts with perceived usefulness to predict sustained engagement.

Additionally, many participants held culturally embedded expectations about the role of a coach, preferring real human interaction and emotional engagement. Such preferences reflect East Asian cultural constructions of the coach as both a technical guide and a moral-emotional figure (Chou and Cheng, 2023), suggesting that AI systems lacking human-like interaction may struggle to establish trust. In system design, incorporating culturally sensitive communication patterns–such as polite verbal feedback or empathetic tone–may help narrow the perceived emotional gap between human and virtual coaches. Policy-level initiatives could also encourage the development of culturally inclusive AI exercise programs that respect local values while promoting digital participation among older populations.

Health limitations also emerged as critical barriers–when users were unable to follow the system’s instructions or receive personalized adjustments, trust and motivation declined (Mitzner et al., 2010). Therefore, accessibility functions such as adjustable exercise intensity, simplified instructions, and safety reminders should be included to accommodate different physical conditions. From a research perspective, future studies could examine how perceived physical competence moderates the relationship between perceived ease of use and behavioral intention.

Goal orientation played a role in perceived usefulness; while some participants aimed to simply maintain health, others sought weight loss or body improvement. Systems that adapt to such individualized goals are more likely to enhance long-term engagement (McMahon et al., 2016). This suggests that goal personalization mechanisms–such as progress tracking dashboards or adaptive goal recommendations–could be valuable in promoting sustained use. Further research may explore how different goal orientations influence motivational persistence among older users of AI coaching platforms.

Finally, some older adults expressed a strong desire for emotional companionship; when AI systems lacked warmth or responsiveness, feelings of loneliness surfaced, underscoring the role of emotional support in technology acceptance among older adults (Wang et al., 2018). This highlights the emotional dimension of human–AI interaction. Consistent with Peng et al. (2022), emotionally responsive and immersive design features may not only mitigate loneliness but also sustain motivation by simulating empathy and a sense of psychological safety. In practical design, incorporating affective feedback elements–such as verbal encouragement, reminders, or adaptive expressions–may help users feel more supported. Future studies could assess whether perceived emotional responsiveness mediates the relationship between trust and continuous usage intention, providing empirical insight into the emotional mechanisms underlying technology acceptance.

#### 5.3.4 Core external variables

Five variables emerged as the most salient and theoretically consequential across the dataset: interface and voice design sensitivity, self-efficacy, technology anxiety, health condition and physical limitation, and emotional companionship need.

Interface and voice design sensitivity repeatedly shaped both PEOU and ATT, revealing that usability among older adults depends not only on operational simplicity but also on emotional approachability. Similarly, self-efficacy and technology anxiety functioned as dual psychological forces–confidence facilitated trust and engagement, while fear inhibited initial adoption. Health condition and physical limitation strongly affected PU, as physical discomfort or inability to follow standardized instructions diminished system credibility and perceived safety. Finally, emotional companionship need was consistently expressed as a decisive affective determinant: even when functional benefits were acknowledged, a lack of warmth or responsiveness led to disengagement. Collectively, these five variables form the conceptual core of an emotionally adaptive TAM applicable to aging contexts, emphasizing that acceptance arises from both cognitive evaluation and affective resonance. The remaining eleven variables demonstrated moderate or situational influence.

The sixteen variables collectively reshape how TAM’s core constructs are conceptualized for older adult populations. PEOU should extend beyond mechanical simplicity to include learning supportiveness and affective accessibility, acknowledging the role of interface empathy and gradual learning mechanisms. PU becomes a dynamic construct, contingent on users’ physical condition, self-efficacy, and personalized health goals rather than a static functional appraisal. ATT, traditionally defined as a rational outcome of PEOU and PU, should now incorporate affective attitude and social presence as key mediators that bridge cognitive trust and emotional acceptance. Together, these theoretical refinements underscore that technology adoption among older adults is neither purely utilitarian nor static, but rather a socially and emotionally embedded process shaped by adaptive interaction between design, cognition, and affect.

## 6 Theoretical implications

This study proposes three key extensions to refine the TAM for aging contexts.

First, technology acceptance should be reconceptualized as a dynamic and adaptive process rather than a static evaluation. Older adults’ perceptions of ease of use and usefulness evolve through repeated interaction, learning, and emotional adjustment. This temporal variability challenges TAM’s assumption of stable cognition, calling for a longitudinal framework that captures how acceptance develops and stabilizes through continuous feedback and self-efficacy growth.

Second, affective, social, and cultural dimensions must be systematically embedded within the model. Emotional resonance, interpersonal expectations, and culturally rooted conceptions of coaching critically shape attitudes toward AI systems. Incorporating constructs such as affective attitude, social presence, and emotional responsiveness can explain why functional utility alone fails to ensure sustained use, particularly in East Asian contexts where relational warmth and respect influence trust.

Third, external variables must be contextualized and empirically validated. This study identifies 16 determinants across technological, psychological, and socio-emotional domains–such as learning pace control, perceived physical competence, and emotional companionship need–that expand TAM’s explanatory scope. These variables provide concrete pathways for future validation through moderation or mediation models using structural equation modeling or longitudinal designs.

In summary, this study advances TAM by situating technology acceptance within the lived realities of aging–characterizing it as a dynamic, socially embedded, and emotionally regulated process. This reconceptualization not only enhances TAM’s theoretical precision but also offers a robust foundation for future empirical testing across diverse older populations.

## 7 Practical implications

Based on the identified contradictions and contextual challenges in older adults’ use of AI fitness coaching systems, this study proposes three key implications.

First, design must be context- and capability-oriented. Older adults’ acceptance depends not only on functional utility but also on emotional interaction, sensory adaptability, and cultural expectations. AI systems should integrate multi-sensory and empathetic design–such as readable interfaces, adjustable speed, and culturally attuned voice feedback–emphasizing learning supportiveness and emotional accessibility as core usability elements.

Second, social trust and participation are essential for sustained adoption. Family encouragement, peer influence, and perceived legitimacy significantly shape older adults’ willingness to use AI technologies. Collaborations among community centers, long-term care institutions, and local governments should promote shared exercise spaces and digital literacy programs that enhance confidence and reduce technology anxiety.

Third, inclusivity in both physical and emotional design is crucial. Systems should accommodate health variability through adaptive difficulty levels, personalized goals, and affective feedback mechanisms that foster motivation and companionship. Policy frameworks are encouraged to establish standards for functional adaptability and emotional responsiveness in AI-based eldercare technologies.

In summary, the value of AI fitness coaches lies not in replacing human trainers but in becoming trusted, adaptive, and emotionally supportive partners that enable older adults to sustain healthy, autonomous lifestyles. These insights bridge the conceptual gaps in TAM by translating cognitive acceptance into emotionally and socially grounded design principles, advancing the development of empathetic and inclusive AI health technologies for aging societies.

## 8 Limitations

This study has several limitations. First, participants’ exposure to the AI fitness coaching system was short-term–limited to a 10-min introduction and a 1-min demonstration. Although this controlled design ensured procedural consistency and minimized COVID-19 contact, it restricted observation of long-term behavioral change, learning curves, and motivation over time. Consequently, the findings reflect initial impressions rather than sustained use. Second, self-selection sampling may have introduced bias, attracting participants already interested in technology or exercise. This limits the representation of those who are skeptical, less active, or digitally inexperienced. Moreover, data collection was confined to urban areas (Taipei and New Taipei City), where digital resources are more accessible, reducing generalizability to rural or lower-income populations. Future research should adopt stratified or purposive sampling to capture diverse age groups, digital literacy levels, and regional or cultural contexts. Longitudinal and repeated-interaction designs are also recommended to examine how perceptions of ease of use, usefulness, and emotional engagement evolve over time.

## 9 Conclusion

Grounded in the TAM, this study explored older adults’ experiences with AI-based fitness coaches through qualitative interviews. The findings reveal that while TAM offers a solid foundation, it insufficiently explains technology use in aging contexts where emotions, bodily conditions, and social meanings play decisive roles. This study advances TAM by reconceptualizing technology acceptance as a dynamic, context-dependent, and emotionally regulated process. Acceptance among older adults is not a one-time decision but a continual negotiation shaped by physical capability, emotional comfort, and social trust. This research integrates external variables, including interface empathy, self-efficacy, health status, and emotional companionship, into the relational structure among perceived ease of use, perceived usefulness, and user attitude. In doing so, it repositions the TAM as a dynamic and interaction-oriented framework, rather than a static evaluative tool. Theoretically, this study introduces and validates the notion of “ambivalent acceptance,” highlighting that older adults’ engagement with AI involves the coexistence of trust and hesitation. Rather than treating emotional fluctuation as error variance, the study reframes it as a psychological mechanism through which older adults negotiate autonomy, safety, and confidence in digital environments. By drawing upon the lived experiences of older adult users, this approach strengthens the ecological validity of TAM and offers a meaningful theoretical advancement by integrating cognitive, emotional, and sociocultural factors that have frequently been neglected in earlier acceptance frameworks. Practically, this study emphasizes that age-friendly AI fitness systems should be designed to promote empathetic interaction, adaptive learning, and relational engagement. The design focus should shift from the question of “Can it be operated?” to “Can it build a meaningful relationship?” This human-centered paradigm broadens the design logic of AI health technologies toward greater inclusivity, sustainability, and emotional trust. Future research should empirically examine these extended variables across different aging populations, such as various age cohorts, levels of digital literacy, and cultural backgrounds, in order to explore how emotional and contextual factors influence technology acceptance pathways within the TAM framework. Longitudinal and cross-cultural studies are especially needed to investigate how trust, self-efficacy, and emotional companionship shape sustained adoption over time. These efforts will help refine the model’s predictive capacity and ensure its applicability to real-world diversity in aging. In conclusion, this study offers a theoretically original and empirically grounded extension of the Technology Acceptance Model by reframing technology acceptance as a socially and emotionally embedded process of “aging with technology.” Its novelty lies in using the lived experiences of older adults as the empirical foundation for model expansion, demonstrating that successful AI health technologies must evolve from tools of efficiency into companions of empathy, empowerment, and relational well-being.

## Data Availability

The original contributions presented in this study are included in this article/supplementary material, further inquiries can be directed to the corresponding author.
